# Selective Elastane Removal Using DMSO–DBN Under Moderate Temperatures: From Pure Filaments to Cotton/Polyester Blends

**DOI:** 10.3390/polym17243247

**Published:** 2025-12-06

**Authors:** Tiago Azevedo, Ana Catarina Silva, Diego M. Chaves, Ana Isabel Ribeiro, Raul Fangueiro, Diana P. Ferreira

**Affiliations:** Centre for Textile Science and Technology (2C2T), University of Minho, 4800-058 Guimarães, Portugal; tiago.azevedo@2c2t.uminho.pt (T.A.); diego.chaves@2c2t.uminho.pt (D.M.C.); afr@2c2t.uminho.pt (A.I.R.); rfangueiro@det.uminho.pt (R.F.)

**Keywords:** elastane degradation, fibre-to-fibre recycling, DMSO, DBN catalysis, textile waste

## Abstract

Selective removal of elastane from textile blends is a critical factor for fibre-to-fibre recycling, since even low elastane content compromises the mechanical shredding efficiency, contaminates recycled streams, and limits the spinnability of recovered fibres. In this work, we investigate dimethyl sulfoxide (DMSO) as a solvent system for elastane degradation under moderate temperatures, both in the absence and in the presence of the organic base catalyst 1,5-diazabicyclo[4.3.0]non-5-ene (DBN). DMSO alone promoted only partial elastane mass loss, typically 26–32% at 80–100 °C (60 min) and up to 79% at 120 °C (10 min), whereas the addition of 0.1% *v*/*v* DBN enabled near-complete or complete mass loss (81–100%) across 80–120 °C within 10–60 min. Complete removal of elastane was achieved in isolated elastane filaments at 100 °C within 30–60 min, and the same treatment conditions were applied to real mixtures of pre-consumer textile waste containing 94% cotton/6% elastane and 87% polyester/13% elastane, leading to permanent dimensional relaxation of the resulting fabrics with area increases of approximately 9–14% and 7–13%, respectively, consistent with the loss of elastane-driven elastic recovery. Scanning electron microscopy (SEM), tensile testing, and dimensional analysis confirmed selective disruption of the elastane, a loss of elastic recovery, and largely preserved morphology and tensile strength of the cotton and polyester fibres. Dimensional change in the treated fabrics served as an indirect indicator of elastane degradation, correlating with the loss of elasticity observed in both blends. In summary, the DMSO–DBN system provides an energy-efficient, controllable, and scalable route for elastane degradation under comparatively mild conditions, thereby contributing to fibre-to-fibre recycling strategies and the advancement of circular textile manufacturing.

## 1. Introduction

Global textile production has increased sharply over the past decades, with recent estimates indicating a total output of approximately 132 million tonnes in 2024 and synthetic fibres accounting for more than two-thirds of this volume. Within this total, elastane represents a relatively small share in terms of mass—around 1.5 million tonnes in 2024, corresponding to about 1.1% of the global fibre market [1]. However, elastane is now incorporated into a very large fraction of apparel products, typically in the range of 2–10 wt% for stretch fabrics, although higher contents may be used in specialised compression or shapewear applications. As a result, a disproportionately high share of post-industrial and post-consumer textile waste contains elastane, even though its overall mass fraction remains modest. A large proportion of this waste consists of multicomponent blends rather than single-fibre textiles, which complicates material recovery and limits the feasibility of fibre-to-fibre recycling, with cotton/elastane and polyester/elastane blends being particularly challenging cases. Elastane (spandex or Lycra^®^) is a segmented polyurethane (PU) whose microphase-separated soft and hard domains provide high elasticity, comfort, and shape retention. Although these properties are desirable in garments across everyday, performance, and medical applications, elastane is problematic from a circularity perspective. Even at low contents, elastane enhances drape, fit, and stretch [1] but introduces substantial barriers to recycling [2] and therefore represents a critical factor to the circularity of cotton- and polyester-based textiles. The intrinsic two-phase architecture—soft segments based on long-chain polyether or polyester diols and hard segments derived from disocyanates and short-chain extenders—confers chemical and mechanical resilience [3]. As a result, elastane in fibre blends can reduce shredding efficiency, promote machine clogging, and diminish the quality and spinnability of recovered fibres. Moreover, elastane is not readily biodegradable and contributes to microplastic pollution, aggravating the environmental burden of elastane-containing textiles [4]. Conventional methods for the removal of elastane from fibre blends have encompassed the utilisation of harsh thermal treatments, the employment of enzymes, and the application of toxic solvents such as dimethylformamide (DMF), dimethylacetamide (DMAc) and tetrahydrofuran (THF) [5,6]. Whilst these methods are effective in solubilizing elastane, they are associated with high energy consumption, toxicity, and strict regulatory limitations. DMF and DMAc, for instance, are classified as hazardous according to the REACH regulations, necessitating the utilisation of specialised equipment for their safe handling and waste management. These drawbacks have prompted the research for more sustainable, selective, and industrially feasible alternatives [7,8,9]. Dimethyl sulfoxide (DMSO), a polar aprotic solvent with a more favourable regulatory/safety profile than DMF and DMAc, has recently gained attention for selective elastane removal. Previous studies show that elastane can be fully dissolved in DMSO at 120 °C within 5–10 min, allowing recovery of the remaining fibres (e.g., PET and PA) while the solvent is recovered and reused at 95–99% [8]. Nevertheless, operation at 120 °C raises energy and process-safety considerations and may affect dyes/finishes or interactions with hydrophilic fibres. Recent work has therefore focused on lowering the operating temperature by combining DMSO with suitable catalysts or co-reagents, while maintaining selectivity towards elastane. Recent advances have explored the combination of DMSO with organic base catalysts to promote elastane degradation under milder conditions [3]. DBN (1,5-diazabicyclo[4.3.0]non-5-ene) is a strong bicyclic amidine base that has been widely used as an organocatalyst for the depolymerisation of polyesters and polyurethanes via base-catalysed cleavage of ester and urethane linkages. In the present work, DBN is combined with DMSO, a polar aprotic solvent that swells the elastane phase, to promote the selective, base-assisted degradation and removal of the segmented polyurethane (elastane) component under comparatively mild conditions. A study reported that the combination of DMSO/DBN with diethylenetriamine enabled the degradation of elastane in cellulosic blends at 80 °C within a 2 h timeframe [3]. This process had a minimal impact on the structural integrity and reusability of the cotton fibres. These and related findings suggest that catalytic DMSO systems could overcome the high temperature barrier associated with conventional solvent-based processes, while maintaining selectivity and preserving the quality of the non-elastane fibres. Notwithstanding these encouraging advancements, there are still considerable challenges to be surmounted if such processes are to be scaled up for industrial applications [5,10,11]. Although less toxic than DMF, DMSO is not entirely innocuous. Its thermal decomposition may be autocatalytic in the presence of strong acids or bases, including DBN, with the potential to result in hazardous exothermic reactions. It is imperative to exercise precise control over reaction conditions and solvent ratios to ensure safety, efficiency, and reproducibility on a large scale. Moreover, economic considerations such as solvent cost, recovery rates, and process integration with existing recycling infrastructure must be addressed. From a circular-economy viewpoint, the feasibility of elastane removal will ultimately depend not only on technical performance, but also on the ability to recover and reuse solvents and to integrate these steps into fibre-to-fibre recycling value chains.

The present study investigates an environmentally friendly degradation strategy for the selective removal of elastane, based on solvent-assisted degradation/disruption in DMSO-based systems enhanced with DBN as a catalyst. The objective of this approach is to encourage the targeted degradation of elastane under moderate thermal conditions (i.e., 80–120 °C), with the aim of enhancing energy efficiency and reducing the environmental impact in comparison with existing solvent-based methods. Our work encompasses systematic evaluations of pure elastane filaments and elastane-containing textiles, combining gravimetric measurements with morphological and chemical changes, and the mechanical properties of the remaining fibres. We first study degradation efficiency and changes in the chemical structure and thermal stability of elastane filaments and then apply the most promising conditions to cotton/elastane and polyester/elastane fabrics. By focusing on solvent-assisted, fibre-selective degradation under milder conditions, this work contributes to the development of sustainable textile recycling technologies that are compatible with emerging circular-economy requirements.

The methodology under discussion has the potential to offer several benefits, including reduced solvent use, lower process temperatures, enhanced fibre preservation, and compatibility with circular economy principles. These developments are of particular pertinence as regulatory pressures and sustainability objectives drive the requirement for scalable, low-impact solutions for the recycling of blended textiles. The outcomes of this research are expected to provide valuable insights for the design of next-generation fibre-to fibre recycling systems, particularly mechanical routes, capable of handling elastane-containing waste, thereby addressing a critical gap in the textile circularity framework.

## 2. Materials and Methods

### 2.1. Materials and Sample Preparation

Commercial elastane yarn (Creora^®^, Hyosung, Seoul, Republic of Korea) was used as the polyurethane (PU) model fibre. The yarn displays a linear density of 47.3 dtex, a tensile strength of 1.15 g/d and an elongation at break of 497%. The pre-consumer textile waste selected for analysis consisted of two binary fabrics with relatively high elastane content, comprising 87% polyester and 13% elastane and 94% cotton and 6% elastane, which were supplied by Riopele–Têxteis, S.A. (Vila Nova de Famalicão, Portugal). Dimethyl sulfoxide (DMSO) and 1,5-diazabicyclo[4.3.0]non-5-ene (DBN) were purchased from Thermo Fisher Scientific (Waltham, MA, USA) and used as received. Untreated fabrics served as controls.

### 2.2. Elastane Degradation

Elastane (EA) degradation assays were conducted using a laboratory beaker dyeing machine (LABIbelus 12 Infra-Red, Pregitzer & Ca., Lda, Guimarães, Portugal) with a bath ratio of 100:1 (20 mL of solution per 0.2 g of elastane). The exhaustion process was applied in both the presence and absence of DBN. Table 1 provides a concise overview of the solvent systems, temperatures, and durations employed in the experimental setup. The elastane yarn was wound around metal supports without the application of tension. The process was repeated with elastane and water alone, in order to ascertain the effect of each chemical agent added. Subsequent to this, the samples underwent a washing step, followed by drying at 40 °C. EA filaments without any treatment were used as control.

The efficacy of the dissolution of elastane was evaluated based on the mass difference observed before and after the treatment (1).

(1) $$Mass\;loss\;\left(\%\right)=100\times \left(1-\frac{mass\;after\;treatment}{mass\;before\;treatment}\right)$$

For conditions in which elastane dissolved, the post-treatment bath was collected and centrifuged at 4000 rpm for 20 min to separate any insoluble residue. The pellet obtained was re-dispersed in distilled water and centrifuged again under the same conditions to promote aggregation and improve phase separation. This washing/centrifugation step was repeated to remove residual solvent. The final sediment (dissolved–reprecipitated elastane fraction and/or degradation products) was collected for further analysis.

Two binary fabrics containing elastane were selected to evaluate both the selective degradation of the elastane component and the potential effect of the treatment on the remaining fibres. These samples corresponded to pre-consumer textile waste and consisted of a polyester/elastane blend (87% polyester/13% elastane) and a cotton/elastane blend (94% cotton/6% elastane). The fabrics were impregnated with the solvents that produced the most favourable results for pure elastane, namely dimethyl sulfoxide (DMSO) in the presence and absence of 1,5-diazabicyclo[4.3.0]non-5-ene (DBN), at temperatures between 80 and 120 °C for different durations. Although the elastane fraction in the cotton blend is relatively low, this composition was intentionally selected to assess the selectivity of the degradation process and its impact on natural fibres, which are predominant in real textile waste streams. The solvent-to-elastane ratio was fixed at 1:100 (*w*/*v*) to ensure complete immersion and uniform reaction, with the solvent volume calculated according to the estimated elastane content in each sample.

### 2.3. Attenuated Total Reflectance-Fourier Transform Infrared Spectroscopy (ATR-FTIR)

Fourier Transform Infrared Spectroscopy (FTIR) coupled with an Attenuated Total Reflectance (ATR) accessory was used to evaluate the influence of the treatment on the elastane surface. The sample analysis was performed using the IRAffinity S1 equipment from Shimadzu (Kyoto, Japan), equipped with an ATR accessory. Spectra were collected over 45 scan cycles and in the spectral range 3800–600 cm^−1^, with a resolution of 4 cm^−1^ in transmittance mode. Three measurements were made on each sample. The intensities of some transmittance peaks were determined by ImageJ 1.54p software to compare the elastane degradation with the different tested methods.

### 2.4. Thermogravimetric Analysis and Differential Thermogravimetric Analysis (TGA/DTG)

Thermogravimetric analysis was performed on STA 7200 Hitachi (Chiyoda, Tokyo, Japan) to assess the thermal stability of the elastane before and after the treatments. TGA plots were obtained within the range of 25–600 °C under a nitrogen atmosphere (200 mL·min^−1^) at 10 °C·min^−1^. The samples were left at room temperature (25 °C) and were placed in an alumina pan. Data were plotted as weight loss (WL) percentage versus temperature, and the mass of dried residues was calculated. The maximum peaks of the thermal transformation events were identified by performing the derivative thermogravimetric analysis.

### 2.5. Scanning Electron Microscopy (SEM)

Scanning Electron Microscopy (SEM) was used to evaluate the surface morphology of elastane and fabrics before and after treatments, using the FlexSEM 1000 II (Hitachi, Tokyo, Japan) scanning electron microscope. The samples were covered with a very thin layer of gold (90 s deposition) via a Quorum MiniQS sputter coater (Hatfield, PA, USA). To capture the secondary electron images, an acceleration voltage of 5 kV was employed.

### 2.6. Mechanical Properties

To evaluate the mechanical properties of the binary fabrics, tensile tests were performed on a Hounsfield H100Ks dynamometer in accordance with the ASTM D5035-11 standard [12], using a 2500 N load cell. Five repetitions were carried out for each treatment on the binary fabric, with the parameters of breaking strength (N), elongation (%) and Young’s modulus (MPa) measured.

## 3. Results and Discussion

This section is organised in two stages: First, we evaluated the controlled degradation and dissolution of elastane filaments in DMSO-based systems, with and without the organic base catalyst DBN, under different thermal conditions. We quantified mass loss and examined the chemical structure (ATR–FTIR), thermal stability (TGA/DTG), and morphology (SEM) of elastane. Second, we applied the most effective solvent–catalyst conditions to real textile waste blends (94% cotton/6% EA and 87% polyester/13% EA) and evaluated fabric-level effects on dimensional stability and mechanical properties.

### 3.1. Elastane Solubilisation Assays

The efficiency of elastane removal was first quantified gravimetrically by comparing the dry mass of elastane filaments before and after treatment, according to Equation (1). Treatments were performed using DMSO, either alone or supplemented with DBN (0.1% *v*/*v*), at temperatures ranging from 80 to 120 °C and exposure times between 10 and 60 min. Based on repeated gravimetric measurements, DMSO alone promoted only partial mass loss under the tested conditions, with values of approximately 26% and 30% after 60 min at 80 °C and 90 °C, respectively, and around 32% at 100 °C (60 min). At 120 °C, mass loss increased to 79% after only 10 min, indicating a strong temperature dependence even in the absence of catalyst. In contrast, the incorporation of DBN led to markedly higher mass loss at intermediate temperatures, with values of about 48% and 65% at 80 °C and 90 °C (60 min), respectively, and complete mass loss (≈100%) at 100 °C (30–60 min) and 120 °C (10 min).

**Figure 1:**
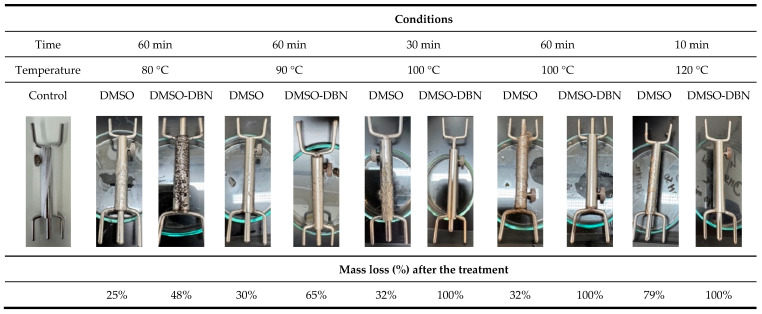
Mass loss of elastane filaments after exposure to DMSO-based solvent systems under different temperature–time conditions, with and without the addition of DBN (0.1% *v*/*v*).

Figure 1 summarises these trends, showing a monotonic increase in mass loss with temperature for both systems and a clearly accelerated response when DBN is present. These results indicate that (i) DMSO alone is capable of partially disrupting elastane at elevated temperature, but (ii) the presence of DBN strongly enhances and accelerates elastane breakdown, enabling full removal at 100 °C within 30–60 min. We interpret “complete mass loss” as effective solubilisation and/or disintegration of the elastane filaments under the applied conditions, consistent with the visual disappearance of intact filaments and with the subsequent recovery of elastane-derived residues from the solvent phase.

### 3.2. ATR-FTIR Analysis

**Figure 2:**
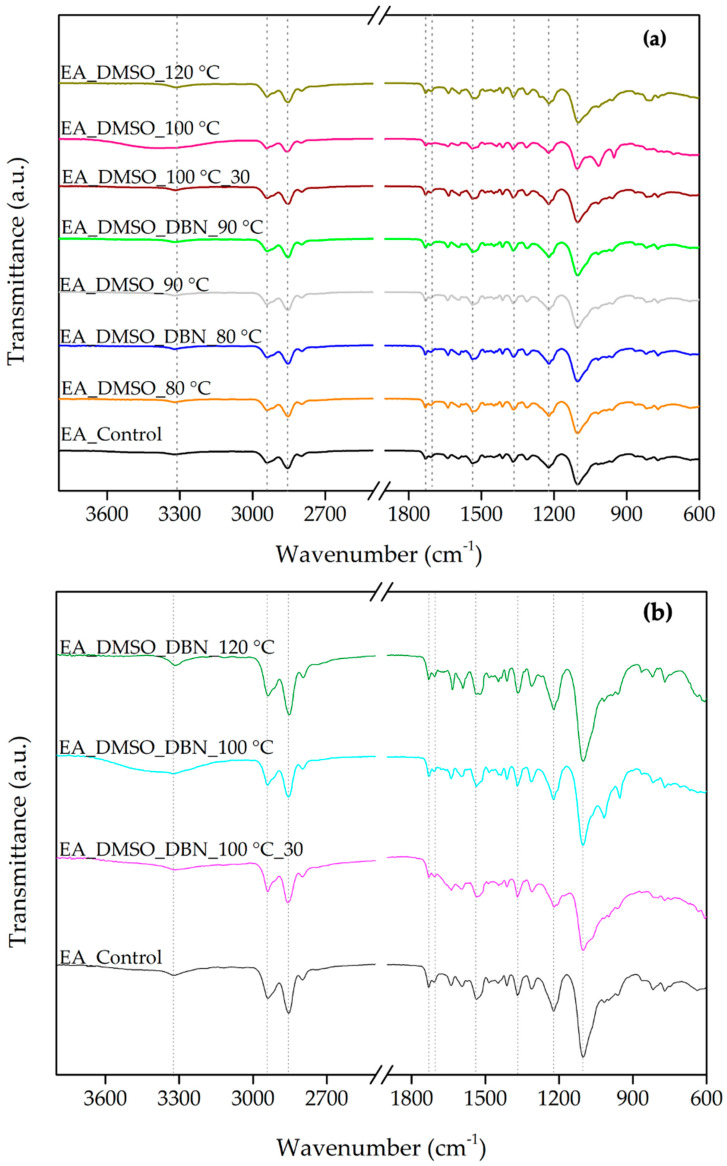
ATR-FTIR spectra of elastane (EA) before and after solvent treatments in DMSO and DMSO + DBN (3800–600 cm^−1^). (**a**) Spectra of untreated EA (control) and EA treated at the indicated temperatures and times; (**b**) Spectra of control and the solid residues recovered by centrifugation from the corresponding treatment baths. Dashed lines indicate the specific wavenumbers of the elastane filaments.

ATR–FTIR spectroscopy was used to examine chemical changes in elastane after treatment and to characterise the solid residues recovered from the post-treatment bath. EA is formed by a copolymer block of polyether-polyurethane through an addition reaction between a polyol (a soft component) and isocyanate groups (a hard component) with hydrogen bonds between the chains, thereby providing structural stability [13,14,15]. In accordance with this hypothesis, the ATR-FTIR spectra exhibited a high degree of similarity with the previously reported spectrum for EA filaments, displaying the characteristic bands from hard and soft segments (Figure 2).

The absorption bands at 3327, 1731, 1537 and 1221 cm^−1^ are attributed to the hard segments of the polyurethane (PU) chain. The absorption bands at 2942, 2854, 1367, and 1102 cm^−1^ are attributed to the soft segments [16,17]. Specifically, the band at 3327 cm^−1^ was attributed to N–H moieties in the urethane groups in the region of hydrogen bonds [16,18]. The bands at 2942 and 2854 cm^−1^ were assigned to C-H asymmetric and symmetric stretching vibrations in the -CH_2_ groups [18]. The band of carbonyl-free groups (C=O stretching) emerged at 1731 cm^−1^ [16,18]. Then, the stretching of hydrogen-bonded carbonyl groups in the crystalline phase of the hard segment domains appeared at 1703 cm^−1^ [16,18]. The band at 1537 cm^−1^ was assigned to the stretching vibration of C=O bending vibration of N-H in the -C-NH group [18]. The bands attributed to C-H in the soft segments emerged at 1367 cm^−1^ [17]. The C-O-C groups appeared at 1221 and 1102 cm^−1^, typical of PU with an ether-based polyol [17,18,19]. The properties of EA strongly depend on hydrogen bonding between N-H and carbonyl or ether oxygen atoms. Some authors have reported that these bonds are reflected in the intensity of the band at 1102 cm^−1^ [16]. Consequently, by comparing the intensity of this band in the FTIR spectra of different samples, a slight decrease can be observed in the sample treated with DMSO at 120 °C, indicating the breaking of hydrogen bonds (Figure 2a).

Moreover, it was reported that the segmental rearrangement and partial cleavage of soft domains also influence the changes in the intensity of this absorption band, while the hydrolysis of urethane bonds affects the changes in the intensity of the bands at 1221, 1703, and 1731 cm^−1^. Therefore, the ratio of intensities of transmittance bands between the 1703 and 1731 cm^−1^ bands and between 1102 and 1221 cm^−1^ bands can be used to compare the EA degradation (Table 2) [20]. The results revealed moderate variations in the intensity ratio between the bands in 1703 and 1732 cm^−1^, associated with the oxidation of soft segments. While the control sample showed a ratio of 0.73, most treated samples presented values in a similar range (0.66–0.76), indicating comparable or slightly increased oxidation levels. A notable exception was observed in the sample treated with DMSO at 100 °C for 30 min, which exhibited a substantially higher ratio of 0.82. This result suggests more extensive oxidative modification of the soft segments under this condition. In contrast, the ratio between the bands at 1102 and 1221 cm^−1^, associated with the hydrolysis of urethane bonds, remained close to the control value of 2.00 in most cases. A modest increase to 2.13 was observed in the sample treated with DMSO at 120 °C, suggesting slightly higher urethane cleavage under harsher thermal conditions. On the other hand, a decrease to 1.89 was recorded for the sample treated with DMSO + DBN at 80 °C, potentially indicating reduced hydrolytic activity under milder conditions or enhanced preservation of urethane bonds in the presence of DBN.

In order to gain further insight into the elastane degradation mechanism, the solid residues recovered from the liquid phase after treatment were isolated and analysed by ATR-FTIR. These residues, hypothesised to be partially solubilised or fragmented elastane components, offer valuable insights into the chemical transformations occurring during the treatments. The FTIR-ATR spectra of the sediments (Figure 2b) exhibited similar characteristic absorption bands for EA filaments, displaying the characteristic bands from both the hard and soft segments. Specifically, the sediment obtained from the treatment at 100 °C for 30 min with DMSO and DBN exhibited a 1703/1732 cm^−1^ ratio of 0.98, which is significantly higher than the control value of 0.73. This marked increase suggests more extensive oxidative modification of the soft segments, likely accompanied by the formation of hydrogen-bonded carbonyls. These findings serve to reinforce the role of DBN as a catalytic agent, thereby enhancing the oxidative degradation pathway in solution. Regarding the 1102/1221 cm^−1^ ratio, associated with the hydrolysis of urethane bonds, the majority of sediment samples exhibited values that were close to or slightly below the control value of 2.00.

Together, these FTIR results support two conclusions. First, DMSO-based treatments, especially at ≥100 °C, induce detectable chemical changes in elastane consistent with disruption of both soft and hard segment organisation. Second, the presence of DBN modifies not only the extent but also the nature of these changes: the ATR-FTIR signature of the recovered sediments indicates that catalytic conditions favour the formation and solubilisation of lower-stability polyurethane fragments, which are then removed from the bulk fibre.

### 3.3. Thermal Analysis

**Figure 3:**
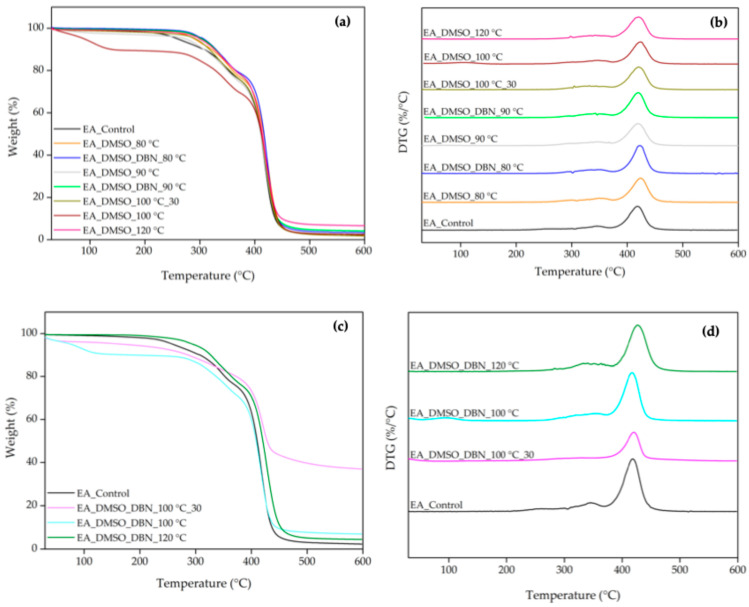
TGA/DTG curves of EA before and after treatments in DMSO and DMSO + DBN, and of the solid residues recovered from the treatment’s baths. (**a**) TGA and (**b**) DTG curves of untreated and treated EA; (**c**) TGA and (**d**) DTG curves of the isolated residues.

Thermogravimetric analysis (TGA) and derivative thermogravimetry (DTG) were used to evaluate how the treatments affected the thermal stability and decomposition profile of elastane (Figure 3, Table 2 and Table 3). In the control sample, decomposition started at approximately 250 °C, with total weight loss at 600 °C and with the highest weight-loss rate at around 383 °C. The TGA/DTG curves revealed three distinct decomposition stages, which can be correlated with the degradation of different structural components of elastane. The initial stage, which occurred at 257 °C, was attributed to the dissociation of the urethane linkages. This was followed by the decomposition of isocyanates at 345 °C. It has been hypothesised that these two steps are associated with the degradation of hard segments in elastane. Subsequently, the third decomposition stage occurred at 418 °C and was attributed to the decomposition of the soft segment (polyol fraction). This stage resulted in significant mass loss (73%), since the soft segment constitutes the majority of EA’s structure (Table 3) [19,21,22].

Following treatments with DMSO and DMSO + DBN, notable changes were observed in the thermal degradation profiles of the elastane samples (Figure 3). In general, the first degradation step—corresponding to the dissociation of urethane linkages, shifted to higher temperatures, ranging from approximately 278 °C to 319 °C. This suggests an increase in the thermal stability of the residual material, likely due to the partial removal of more labile components during treatment. In several samples, a minor mass-loss event appeared below 120 °C, probably related to residual solvent evaporation or low-molecular-weight by-products of degradation. The most pronounced differences were detected in the third degradation stage, which corresponds to the breakdown of the soft polyol-rich segments. Compared to the control (73% mass loss at 418 °C), treated samples showed reduced mass loss values ranging from 47% to 69%, indicating partial dissolution or structural disruption of these domains. The sample treated with DMSO at 80 °C exhibited the most significant reduction (47%), suggesting substantial depletion of soft-segment-rich material even under milder, non-catalytic conditions. In contrast, samples treated with DMSO in the presence of DBN displayed more complex behaviours. For instance, the treatment at 80 °C with DBN showed a soft-segment loss similar to the control (~75%), suggesting either incomplete degradation or possible re-precipitation of degraded fragments. At 100 °C with DBN, the corresponding high-temperature mass loss decreased to 65.2%, consistent with more effective depletion of the soft-segment component under catalytic conditions. Additional insights were obtained by analysing the sediment recovered after dissolution. The TGA profiles of these solid residues (Figure 3b) revealed lower onset degradation temperatures compared with the EA_control with initial mass-loss events as low as 93 °C and 134 °C in the samples treated with DMSO + DBN at 100 °C. This thermal instability suggests that these sediments are composed of oxidised and fragmented elastane components, consistent with significant polymer scission occurred during treatment.

To further compare stability, characteristic temperatures for 5%, 10% and 20% weight loss (T_5%_, T_10%_, T_20%_) and an onset temperature for the main degradation regime (T_onset_) were extracted. Generally, treated elastane samples showed higher T_5%_, T_10%_, T_20%_ and T_onset_ than the untreated control (Table 4). This trend indicates an increase in the thermal stability of the residual material, likely resulting from selective degradation or removal of the more labile soft segments. In particular, treatments such as DMSO + DBN at 80 °C and 90 °C showed consistent elevation across all degradation thresholds, suggesting a relative enrichment in thermally stable hard segments. Interestingly, despite being subjected to aggressive conditions, the sample treated with DMSO at 100 °C without time restriction exhibited the highest onset temperature (395 °C), potentially reflecting extensive soft-segment degradation and the preservation of a more stable residue. These results highlight the distinct impact of each treatment on the soft/hard segment balance [23] in the elastane structure, and corroborate the effectiveness of DMSO-based systems, especially in combination with DBN, in modulating the degradation pathway and thermal behaviour of elastane [23].

Overall, the TGA-DTG data corroborate the ATR-FTIR findings, validating that the DMSO-based treatments—particularly those employing DBN—promote base-catalysed cleavage of urethane and ester linkages within the elastane polyurethane. DBN is expected to act as a strong Brønsted base, deprotonating available hydroxyl or N–H sites (e.g., chain-end polyols, urethane NH groups or traces of water), and generating nucleophilic alkoxide/amide species. These anions can then attack the carbonyl carbon of urethane and soft-segment ester groups, leading to C–O bond scission and formation of lower-molar-mass polyurethane fragments, while DBN is regenerated after proton transfer, consistent with its catalytic role. These effects are especially evident at 100 °C, where the catalytic treatment achieved results comparable to harsher conditions, but with lower thermal input. The preservation of the characteristic urethane and ether bands in the recovered sediments, combined with their reduced thermal stability, suggests that these residues are composed of chemically modified elastane-derived oligomers, in which both soft and hard segments have undergone partial oxidation and bond cleavage, rather than entirely new small-molecule species. We note that the present work does not directly identify individual degradation products (e.g., by LC-MS or NMR); therefore, the mechanism discussed above should be regarded as a plausible catalytic pathway consistent with the literature and our indirect structural/thermal evidence, rather than a fully resolved reaction scheme.

### 3.4. SEM Analysis

**Figure 4:**
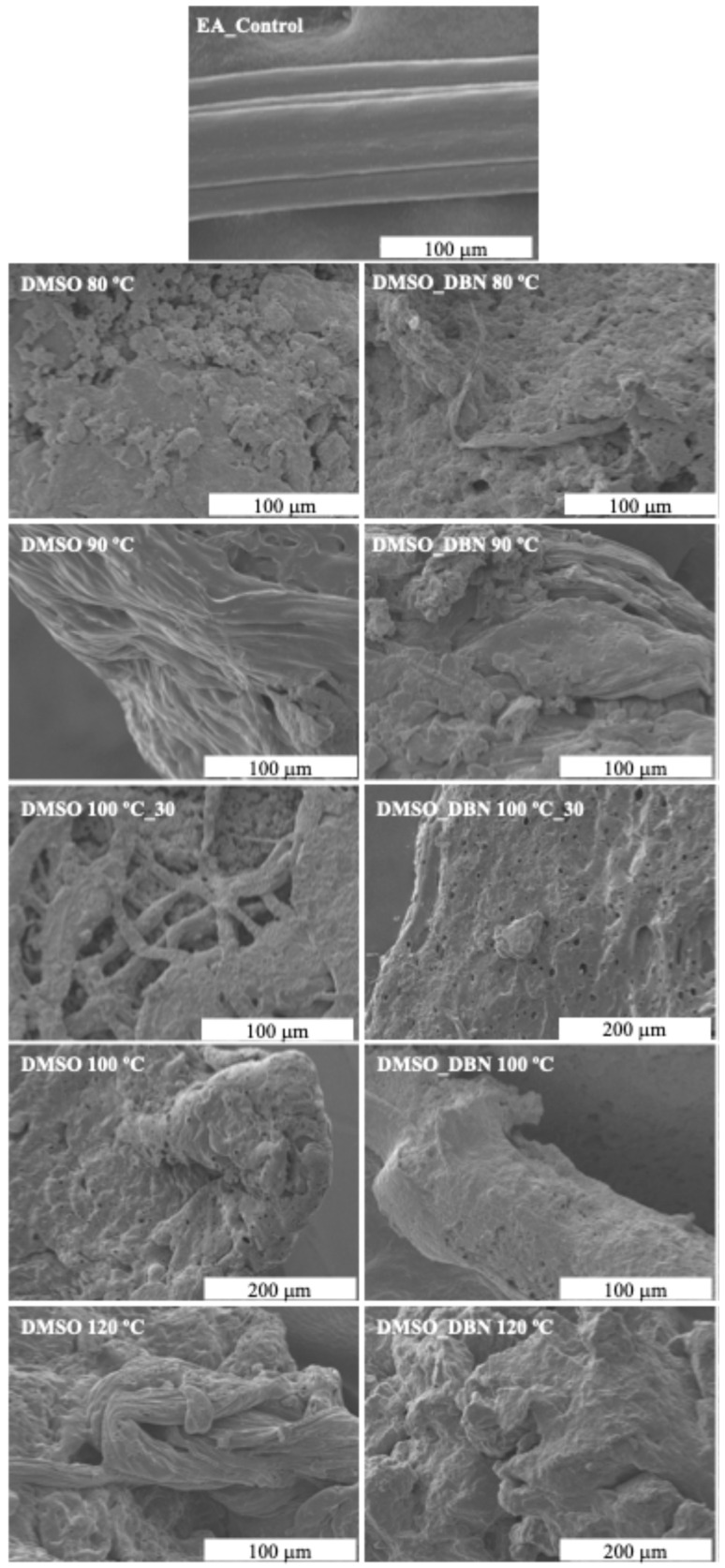
SEM micrographs of elastane (EA) filaments before and after treatment in DMSO-based systems.

Scanning electron microscopy (SEM) images were collected to evaluate the effectiveness of EA filaments degradation and the corresponding morphology modifications after the treatments, using elastane without any treatment as control (Figure 4). The SEM images revealed the structure of the EA filaments, which shows longitudinal striations that resemble a multifilament structure due its irregular cross-section. The untreated control sample (EA_control) exhibited a smooth and homogeneous surface; however, treatments with DMSO alone resulted in progressive surface disruption and fibre collapse, with increasing severity from 80 °C to 120 °C. As the temperature was increased, the morphology became more amorphous, indicating substantial structural breakdown. A more pronounced surface erosion, pore formation and filament break-up were observed in the presence of DBN at all temperatures tested. SEM images revealed more severe morphological changes at each temperature, with earlier onset of fibre disintegration, pore formation, and aggregation. These observations suggest that there is enhanced elastane dissolution and polymer chain rearrangement, which is promoted by the catalytic action of DBN. The results of the SEM analysis demonstrate that DMSO-based treatments, particularly in the presence of DBN, are effective in disrupting the structural integrity of elastane, supporting the proposed solvent-assisted, catalyst-enabled degradation pathway.

### 3.5. Case Study: Elastane Degradation from Cotton/Elastane and Polyester/Elastane Textile Blends

To assess the selective degradation of elastane in real pre-consumer textile waste blends, two representative fabrics, 94% cotton (CO)/6% elastane (EA) and 87% polyester (PES)/13% elastane (EA), were treated with DMSO, both with and without the organic base catalyst DBN. A solvent-to-elastane ratio of 100:1 was used to define the required treatment volume.

In order to evaluate the structural impact of the treatments, mechanical tests were conducted to measure dimensional variation, tensile strength and elongation. The dimensional variation test was performed to evaluate the influence of the treatments on the elastic recovery of the material. To this end, the dimensions of the samples were measured (Equation (2)), and their areas (cm^2^) were calculated before and after treatment with DMSO and DBN under the different conditions mentioned above.

(2) $$Dimensional\;variation\left(\%\right)=\left(\frac{Initial\;area-Final\;area}{Initial\;area}\right)\times 100$$

#### 3.5.1. Cotton/Elastane Blend

**Figure 5:**
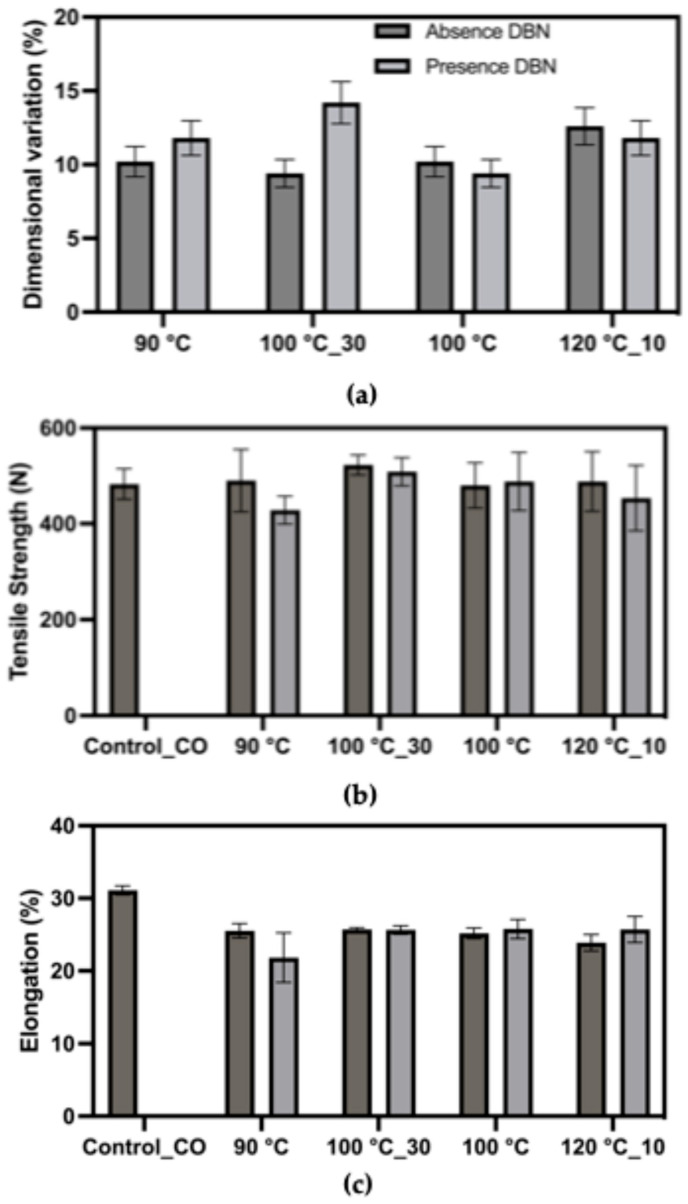
Mechanical response of the cotton/elastane fabric (94% cotton/6% elastane) after treatment in DMSO and DMSO + DBN at different temperatures and times. Plotted parameters include: (**a**) dimensional variation (%) relative to the initial fabric area, used as an indirect indicator of the loss of elastic recovery after elastane removal; (**b**) tensile strength (breaking force, N); (**c**) elongation at break (%).

As shown in Figure 5, dimensional variation values ranged from approximately 9% to 14% across treated samples, relative to an initial area of 169 cm^2^, with generally higher variation observed in the presence of DBN. The sample treated at 100 °C for 30 min with DBN exhibited the highest dimensional variation, suggesting significant disruption of elastane functionality under this condition. Tensile strength of the treated fabrics remained within a relatively stable range (420–480 N), indicating that the structural integrity of the cotton fibres was largely preserved despite chemical treatment. However, minor strength reductions were observed in certain conditions, particularly at 90 °C, suggesting mechanical integrity was largely preserved. Elongation at break showed moderate variation, with most treated samples exhibiting values between 20% and 25%. The lowest elongation was observed in the 90 °C + DBN treatment, which may indicate partial stiffening or greater degradation of elastane. Notably, the elongation trend did not follow a clear correlation with temperature, suggesting complex interactions between the treatment conditions and fabric morphology.

#### 3.5.2. Polyester/Elastane Blend

**Figure 6:**
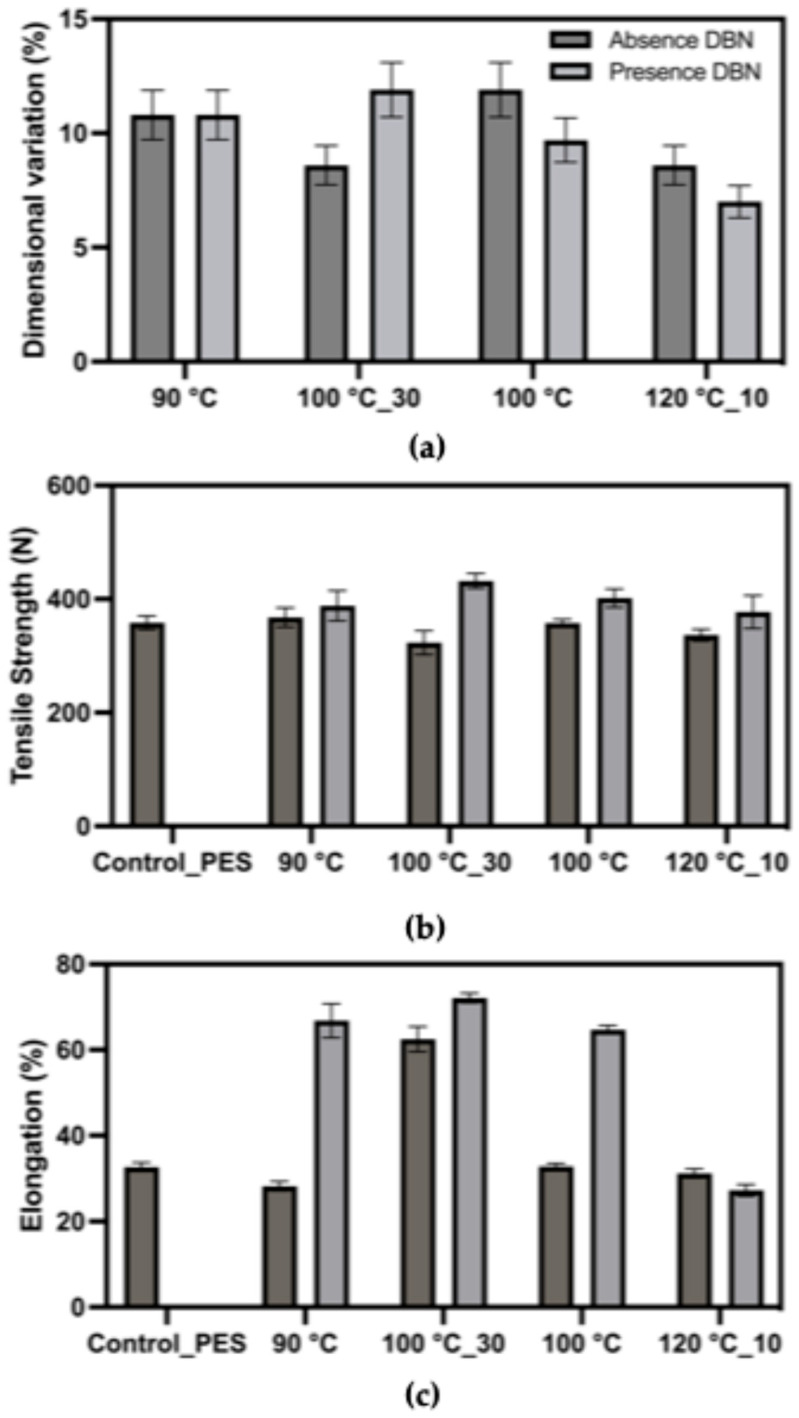
Mechanical response of the polyester/elastane fabric (87% polyester/13% elastane) after treatment in DMSO and DMSO + DBN at different temperatures and times. Plotted parameters include: (**a**) dimensional variation (%) relative to the initial fabric area (loss of elastic recovery); (**b**) tensile strength (breaking force, N); (**c**) elongation at break (%).

In the polyester/elastane fabric (Figure 6), dimensional variation values were slightly lower overall, ranging from 7% to 13%, relative to an initial area of 361 cm^2^. Treatments at 100 °C, both with and without DBN, resulted in the highest variation, again indicating notable alteration to the fabric’s elastic response. The addition of DBN did not systematically increase variation, suggesting different solvent-fibre interactions compared to the cotton blend. Tensile strength remained relatively consistent across treatments, with most values clustering around 320–380 N. This suggests that polyester fibres maintained their mechanical resistance throughout the degradation process, in line with their known chemical stability. Elongation values varied more widely than in the cotton-based fabric, reaching up to 60% in samples treated at 90 °C and 100 °C with DBN, indicating that, after treatment, deformation proceeds through plastic extension rather than classic elastane-driven elastic recovery. In contrast, samples treated at 120 °C or without DBN generally showed lower elongation, suggesting more effective elastane disruption or morphological compaction.

The results demonstrate that DBN plays a significant role in enhancing elastane degradation, particularly at moderate temperatures such as 100 °C. Treatments with DBN tend to increase dimensional variation and affect elongation more markedly, supporting its catalytic role in promoting fibre disintegration. However, tensile strength values remained generally stable, suggesting that both cotton and polyester backbones were not significantly compromised under the tested conditions. In the context of textile recycling, the use of DBN in DMSO-based systems appears to offer a viable strategy for selectively targeting elastane filaments while preserving the primary fibre structure. These findings reinforce the potential of milder degradation conditions (e.g., 100 °C for 30 or 60 min) as energy-efficient alternatives to harsher protocols, provided that a catalyst is used to facilitate the reaction.

**Figure 7:**
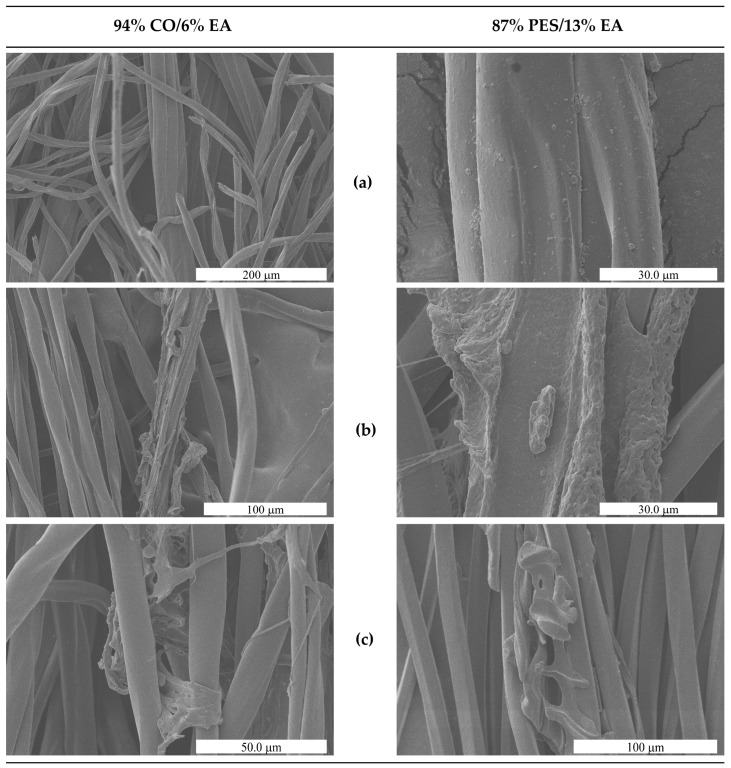
SEM micrographs of the two elastane-containing fabrics before and after solvent treatment. (**a**) Untreated fabrics, showing intact elastane filaments embedded within the cotton or polyester fibre network. (**b**) Fabrics after treatment in DMSO at 100 °C for 30 min. (**c**) Fabrics after treatment in DMSO + DBN (0.1% *v*/*v*) at 100 °C for 30 min.

Scanning Electron Microscopy analysis of treated fabric surfaces (Figure 7) supported these mechanical observations. In both cotton/elastane (94% CO/6% EA) and polyester/elastane (87% PES/13% EA) fabrics, treatment with DMSO + DBN at 100 °C for 30 min led to visible depletion or fragmentation of elastane filaments, while the surrounding cotton or polyester fibres remained intact and retained their characteristic morphology. The disappearance or collapse of elastane-rich domains, combined with the preserved appearance of the non-elastomeric fibres, is consistent with a selective removal process.

Overall, these results demonstrate that DMSO-based systems, particularly in the presence of DBN, can (i) disrupt and remove the elastane phase under moderate thermal conditions (down to 100 °C for 30–60 min), (ii) induce a measurable loss of elastic recovery in commercial fabrics (dimensional relaxation), and (iii) preserve the load-bearing capacity of the cotton and polyester matrices. This provides direct experimental evidence that solvent-assisted, catalyst-enabled elastane removal is feasible in real textile blends and supports downstream fibre recovery for circular recycling.

An important limitation of the present work is that solvent and catalyst recovery were not experimentally addressed. The overall sustainability, process scalability and practical relevance of the proposed route will ultimately depend on the ability to recover and reuse DMSO and DBN in a closed-loop system. DMSO is already employed in processes where solvent recovery by distillation and reuse is technically feasible, which supports its potential integration into circular schemes [24,25,26]. However, a dedicated assessment of DMSO/DBN recyclability in the specific context of elastane removal, including solvent recovery efficiency, DBN stability and life-cycle impacts, remains necessary and is planned for future work.

## 4. Conclusions

This study demonstrates that DMSO-based solvent systems—particularly when combined with the organic base catalyst DBN—can selectively disrupt and remove the elastane phase under moderate thermal conditions (≤120 °C) through solvent-assisted degradation of the segmented polyurethane structure. In elastane filaments, DMSO + DBN at 100 °C for 30–60 min (and at 120 °C for 10 min) led to near-complete to complete elastane removal. When applied to pre-consumer waste fabrics of cotton/elastane (94/6) and polyester/elastane (87/13), the treatments induced permanent dimensional relaxation consistent with the loss of elastane-driven elastic recovery, while the morphology and tensile strength of the cotton and polyester matrices were largely preserved. ATR-FTIR, TGA/DTG, and SEM together corroborate selective disruption of elastane domains and the formation/removal of lower-stability fragments. Overall, these results indicate the technical feasibility of elastane removal at moderate temperatures under the laboratory conditions employed and support downstream fibre recovery in fibre-to-fibre recycling schemes. The proposed treatment route offers a promising step toward improving the recyclability of elastane-containing textiles, enabling more effective separation of blended materials within circular textile value chains. Future work will focus on molecular-level identification of the degradation products and on integrating this treatment with solvent recovery and fibre-to-fibre recycling schemes. In addition, we will investigate molecular weight and crystallinity changes in the different fibre components in order to fully resolve the structural impact of the treatment, and we will assess closed-loop solvent management, including DMSO recovery and DBN reuse, as a key step towards evaluating the full environmental and industrial viability of the proposed elastane removal process.

## Figures and Tables

**Table 1:** Methodologies to follow for the solubilization of pure elastane.

Method	Conditions	Reagents
Exhaustion	80, 90 and 100 °C, 1 h 100 °C 30 min 120 °C, 10 min	DMSO with presence or absence of DBN (0.1% *v*/*v*) Bath ratio 100:1

DMSO: Dimethyl sulfoxide; DBN: 1,5-Diazabicyclo[4.3.0]non-5-ene.

**Table 2:** The ratio of transmittance intensities in the FTIR spectra of elastane (EA) samples analysed between the absorption bands at 1703 and 1732 cm^−1^ indicating the oxidation of soft segments of EA, and the bands at 1102 and 1221 cm^−1^ indicating the hydrolysis of urethane bonds in the EA.

Sample	*I*_1703/1732_ Oxidation of Soft Segments	*I*_1102/1221_ Hydrolysis of Urethane Bonds
EA_Control	0.73	2.00
EA_DMSO 80 °C	0.75	2.00
EA_DMSO_DBN 80 °C	0.66	1.89
EA_DMSO 90 °C	0.66	1.97
EA_DMSO_DBN 90 °C	0.71	2.00
EA_DMSO 100 °C_30	0.82	1.93
EA_DMSO 100 °C	0.76	1.97
EA_DMSO 120 °C	0.74	2.13
Sediment		
EA_DMSO_DBN 100 °C_30	0.98	1.90
EA_DMSO_DBN 100 °C	0.72	2.09
EA_DMSO_DBN 120 °C	0.86	1.94

**Table 3:** TGA results of untreated and treated elastane samples in terms of degradation peaks (DP) and corresponding temperature and weight loss (WL).

Samples	DP	T (°C)	WL (%)
EA_Control	1	257	8.50
	2	345	16.1
	3	418	73.0
EA_DMSO 80 °C	1	300	19.7
	2	349	14.3
	3	424	47.3
EA_DMSO + DBN 80 °C	1	298	4.43
	2	349	16.4
	3	422	75.6
EA_DMSO 90 °C	1	78	2.86
	2	296	7.63
	3	345	14.8
	4	419	71.4
EA_DMSO + DBN 90 °C	1	297	5.29
	2	343	17.5
	3	420	72.9
EA_DMSO 100 °C_30	1	304	9.05
	2	332	15.7
	3	420	69.3
EA_DMSO 100 °C	1	107	10.3
	2	302	7.88
	3	348	13.8
	4	424	65.2
EA_DMSO 120 °C	1	298	1.44
	2	342	16.3
	3	420	71.1
Sediments			
EA_DMSO_DBN 100 °C_30	1	134	1.26
	2	317	12.2
	3	412	45.7
EA_DMSO_DBN 100 °C	1	93	8.04
	2	354	19.6
	3	420	63.4
EA_DMSO_DBN 120 °C	1	274	3.06
	2	332	19.5
	3	417	70.6

**Table 4:** TGA results of untreated and treated elastane (EA) samples in terms of temperature with weight loss of 5% (T_5%_), 10% (T_10%_), and 20% (T_20%_), and onset temperature (T_onset_).

Sample	T_5%_ (°C)	T_10%_ (°C)	T_20%_ (°C)	T_onset_ (°C)
EA_Control	259.8	308.2	352.8	381
EA_DMSO 80 °C	289.7	321.3	359.9	382
EA_DMSO_DBN 80 °C	306.4	329.2	365.9	389
EA_DMSO 90 °C	272.6	307.2	349.9	384
EA_DMSO_DBN 90 °C	302.9	327.8	364.1	379
EA_DMSO 100 °C_30	291.8	317.4	354.4	383
EA_DMSO 100 °C	89.0	139.1	328.2	395
EA_DMSO 120 °C	299.1	326.3	364.7	384
Sediments				
EA_DMSO_DBN 100 °C_30	165.7	278.9	363.8	376
EA_DMSO_DBN 100 °C	74.1	180.6	338.7	385
EA_DMSO_DBN 120 °C	286.7	318.3	355.2	381

## Data Availability

The original contributions presented in this study are included in the article. Further inquiries can be directed to the corresponding authors.

## Abbreviations

The following abbreviations are used in this manuscript:

DMSO	Dimethyl sulfoxide
DBN	1,5-diazabicyclo[4.3.0]non-5-ene
EA	Elastane
PES	Polyester
CO	Cotton
SEM	Scanning electron microscopy
DMF	Dimethylformamide
DMAc	Dimethylacetamide
PU	Polyurethane
ATR-FTIR	Attenuated total reflectance-Fourier transform infrared spectroscopy
TGA/DTG	Thermogravimetric analysis and differential thermogravimetric analysis
