# Correction: Azevedo et al. Selective Elastane Removal Using DMSO–DBN Under Moderate Temperatures: From Pure Filaments to Cotton/Polyester Blends. *Polymers* 2025, *17*, 3247

**DOI:** 10.3390/polym18091060

**Published:** 2026-04-28

**Authors:** Tiago Azevedo, Ana Catarina Silva, Diego M. Chaves, Ana Isabel Ribeiro, Raul Fangueiro, Diana P. Ferreira

**Affiliations:** Centre for Textile Science and Technology (2C2T), University of Minho, 4800-058 Guimarães, Portugal; tiago.azevedo@2c2t.uminho.pt (T.A.); diego.chaves@2c2t.uminho.pt (D.M.C.); afr@2c2t.uminho.pt (A.I.R.); rfangueiro@det.uminho.pt (R.F.)

Ana Isabel Ribeiro was not included as an author in the original publication [1]. The corrected Author Contributions statement appears here.

**Author Contributions:** Writing—original draft preparation, A.C.S. and T.A.; writing—review and editing, R.F. and D.P.F.; conceptualization, D.P.F., A.I.R. and A.C.S.; methodology, A.I.R. and T.A.; validation, R.F. and D.P.F.; formal analysis, A.C.S., A.I.R. and T.A.; investigation, A.C.S., A.I.R., T.A. and D.M.C.; resources, D.P.F. and R.F.; data curation, D.M.C.; visualisation, D.M.C.; supervision, D.P.F., A.I.R. and A.C.S.; project administration, D.P.F. and R.F.; funding acquisition, D.P.F. and R.F. All authors have read and agreed to the published version of the manuscript.

The authors state that the scientific conclusions are unaffected. This correction was approved by the Academic Editor. The original publication has also been updated.
